# Two Case Reports of Neuroinvasive West Nile Virus Infection in the Critical Care Unit

**DOI:** 10.1155/2012/839458

**Published:** 2012-08-27

**Authors:** Edgardo M. Flores Anticona, Hadeel Zainah, Daniel R. Ouellette, Laura E. Johnson

**Affiliations:** ^1^Internal Medicine Department, Henry Ford Health System, Wayne State University School of Medicine, 2799 West Grand Boulevard, CFP1, Detroit, MI 48202, USA; ^2^Infectious Diseases Division, Henry Ford Health System, Wayne State University School of Medicine, 2799 West Grand Boulevard, CFP 304, Detroit, MI 48202, USA; ^3^Pulmonary and Critical Care Division, Henry Ford Health System, Wayne State University School of Medicine, 2799 West Grand Boulevard, Detroit, MI 48202, USA

## Abstract

We describe the clinical course of two cases of neuroinvasive West Nile Virus (WNV) infection in the critical care unit. The first case is a 70-year-old man who presented during summer with mental status changes. Cerebrospinal fluid (CSF) analysis revealed pleocytosis with lymphocyte predominance. WNV serology was positive in the CSF. His condition worsened with development of left-sided weakness and deterioration of mental status requiring intensive care. The patient gradually improved and was discharged with residual left-sided weakness and near-complete improvement in his mental status. The second case is an 81-year-old man who presented with mental status changes, fever, lower extremity weakness, and difficulty in walking. CSF analysis showed pleocytosis with neutrophil predominance. WNV serology was also positive in CSF. During the hospital stay his mentation worsened, eventually requiring intubation for airway protection and critical care support. The patient gradually improved and was discharged with residual upper and lower extremity paresis. Neuroinvasive WNV infection can lead to significant morbidity, especially in the elderly. These cases should be suspected in patients with antecedent outdoor activities during summer. It is important for critical care providers to be aware of and maintain a high clinical suspicion of this disease process.

## 1. Introduction

West Nile Virus (WNV) could be of significant morbidity and mortality; high clinical suspicion should be present to diagnose this disease. Neuroinvasive WNV might be severe enough to require critical care. In this paper, we describe two cases of neuroinvasive WNV.

## 2. Virology

WNV is an RNA arbovirus, a member of the *Flaviviridae* family. The *Flavivirus* genus also includes other human pathogens such as dengue, yellow fever, and Japanese encephalitis viruses.

West Nile virions are spherical and enveloped with a diameter of 50 nm and icosahedral symmetry. The genome consists of ribonucleic acid [1]. Phylogenic studies based on sequences of the WNV genome identified 5 lineages of WNV. The virus that entered North America belongs to lineage I, this lineage is also found in Europe and Africa and the Middle East. Lineage II is mainly of African origin. The relevance of this is that, in general, lineage I (except for the Australian strain, clade Ib) can cause severe neurological disease, whereas lineage II and lineage I (clade Ib) are associated with mild, self-limited disease [2, 3].

## 3. Transmission

The transmission of the disease in humans occurs most commonly from a bite of an infected mosquito. The natural cycle of WNV is maintained in nature by many species of mosquitoes and birds. Crows, magpies, jays, house sparrows, house finches, and grackles are effective reservoirs for infected mosquitoes. Among mosquitoes, *Culex* species plays the most important role in natural transmission [3]. Humans and other mammals, especially horses, are incidental hosts and do not play a role in the transmission cycle since they do not develop sufficiently prolonged or high-level viremia [3, 4]. Human infection occurs predominately through infected mosquitoes that acquired the virus from infected hosts, primarily birds. Person-to-person transmission is not common but can result from blood transfusion, organ transplantation, intrauterine or breastfeeding exposure [5]. In the second case, there was an antecedent event of outdoor activities, specifically hiking 2 weeks prior to presentation. In both cases, the exposure was in the summer, which is consistent with the seasonal outbreaks of WNV infection.

## 4. Clinical Manifestations

The incubation period of WNV is 2–15 days. More than 80% of the people infected with WNV will not develop any symptoms. Approximately 20% will develop a self-limited flu-like illness called West Nile fever. West Nile fever is characterized by fever, myalgias, headaches, gastrointestinal disturbances, and a maculopapular rash that is present in one-third to one-half of cases. There is no nervous system involvement in West Nile fever [6].

Less than 1% of infected persons develop neurological disease. Neuroinvasive disease occurs when the virus penetrates the blood-brain barrier and invades certain groups of neurons, particularly in the brainstem, deep nuclei, and anterior horn of the spinal cord [7]. The clinical presentation of the neuroinvasive disease varies and includes encephalitis, aseptic meningitis, and poliomyelitis-like syndrome [8]. For patients with neuroinvasive disease, 35–40% have meningitis, 55–60% have encephalitis, and only 5–10% have poliomyelitis; presentation depends on the area or season. Overlap syndromes can also occur [7].

The presentation of encephalitis is similar to other viral encephalitis with fever, headaches, and altered mental status. Other symptoms like diarrhea, vomiting, and rash are also seen. A prominent finding in West Nile encephalitis is muscular weakness (30–50% of patients with encephalitis), often with lower motor neuron symptoms, flaccid paralysis, and hyporeflexia with no sensory abnormalities [8]. Other manifestations of encephalitis include cranial nerve involvement (especially the facial nerve) and movement disorders (postural or kinetic tremor and parkinsonism). West Nile encephalitis has a predilection for the elderly. In our case-series, both patients were older, and had encephalitis with remarkable motor deficit in different extremities, in addition to worsening hand tremors in the second case. Also, both patients required intubation and intensive care due to worsening mentation.

West Nile poliomyelitis is characterized by flaccid paralysis syndrome that can be isolated or occurs along with encephalitis or meningitis. This form of presentation has an acute onset and rapid progression of asymmetric flaccid weakness with hyporeflexia or areflexia of the affected limbs [8]. Weakness can occur in a single limb or in any combination of the four extremities. Guillain-Barré syndrome and generalized myeloradiculitis have also been reported [4].

Respiratory failure requiring endotracheal intubation may also occur in up to 38% in one series [9]. Other less common presentations associated with WNV include fulminant hepatitis [9], pancreatitis [10], myocarditis, cardiac arrhythmias, myositis, orchitis [11], nephritis, optic neuritis, [4] and hemorrhagic fever with coagulopathy [9].

## 5. Case Description

### 5.1. Case 1

A 70-year-old man with a history of hypertension and coronary disease presented in July with a 2-day history of mild-to-moderate headache and gradual change in his mental status. Initial physical examination revealed changes in his mentation. The patient was alert and oriented but displayed subtle personality changes. The physical examination was otherwise unremarkable including the rest of the neurological examination. Cerebrospinal fluid (CSF) analysis revealed pleocytosis (419 cells/*μ*L) with lymphocyte predominance (66%), increased protein (93 mg/dL), and normal glucose (69 mg/dL). The patient was started on empiric antimicrobials for potential bacterial and herpetic meningitis (with vancomycin, ceftriaxone, ampicillin, and acyclovir). Upon admission, blood and urine cultures were negative; initial bacterial and viral analysis of the CSF was also negative. The patient developed fever 4 days after the onset of the symptoms. His mental status continued to deteriorate requiring endotracheal intubation and subsequent admission to the intensive care unit (ICU). He also developed left-sided weakness. Electroencephalogram revealed mild-to-moderate encephalopathy with no epileptic activity. Brain magnetic resonance imaging (MRI) was consistent with chronic ischemic changes and revealed nonspecific signals within the middle cerebellar peduncle bilaterally. The patient continued to be febrile without an evident source of infection. Eleven days later, WNV immunoglobulin (Ig) M was positive in the CSF (titers of 1 : 8) establishing the diagnosis of West Nile encephalitis. The hospital course was complicated by ventilator-associated pneumonia and urinary tract infection, both treated appropriately. The patient failed numerous extubation attempts, which necessitated tracheostomy. In addition, he required feeding tube placement. The patient was discharged to an intermediate care facility after a prolonged hospitalization that lasted 42 days, including 40 days in the ICU. Upon discharge, the patient had residual left-sided weakness and near-complete improvement in his mental status.

### 5.2. Case 2

An 81-year-old man with a history of hypertension, diabetes, atrial flutter, and sick sinus syndrome presented to the hospital in July complaining of a 3-day history of fever, chills, rigors, myalgia, worsening hand tremors, lower extremity weakness, and difficulty in walking. Two weeks prior, he was feeling well and went hiking. Two days prior to admission, family members noted a progressive change in his mental status. The initial assessment revealed disorientation, intentional tremors, and weakness in the left lower extremity. CSF analysis showed pleocytosis (24 cells/*μ*L) with neutrophil predominance (81%), increased protein (68 mg/dL), normal glucose (64 mg/dL), and negative Gram stain. The patient was empirically treated for bacterial and viral meningitis with acyclovir, ceftriaxone, ampicillin, and vancomycin. Blood and CSF cultures were negative. Furthermore, other CSF tests were negative for varicella, herpes, syphilis, Epstein-Barr virus, *Cytomegalovirus*, and *Cryptococcus*. Electroencephalogram showed moderate encephalopathy with no epileptiform activity. Head tomography was normal; however, brain MRI was not obtained due to intracardiac hardware. The patient continued to have fever, rigors, and declining mental status. Additionally, the motor impairment progressed to involve the upper extremities. Five days later, he was transferred to the ICU due to respiratory distress. Health-care-associated pneumonia was diagnosed and treated accordingly. By that time (day 7), CSF serology revealed positive WNV IgM (titers of 1 : 3.83). On day 14, the patient was intubated for 7 days secondary to worsening mentation. The clinical course was also complicated by upper gastrointestinal bleed. The patient gradually improved and was discharged with residual upper and lower extremities paresis. Overall, the patient was hospitalized for 33 days including 15 days in the ICU.

This case series was prepared by clinicians directly involved in the patient's care and provided no identifiable personal health information. The patients were agreeable to this paper.

## 6. Discussion

West Nile virus was first isolated in 1937 from the blood of a woman with febrile disease who lived in the West Nile region of Uganda [12]. This mosquito-borne disease is endemic in Africa, Middle East, and southern Asia and has recently spread to Europe and North America [13, 14]. The first cases of WNV in the United States were reported in New York in 1999 when an outbreak took the lives of 7 people while 62 suffered from neuroinvasive disease [15, 16]. Sequence analysis of the virus from the initial cases suggested that the virus may have been imported from the Middle East [15, 17]. WNV rapidly spread resulting in the largest epidemic of neuroinvasive disease in US in 2002 to 2003. Since its introduction, WNV has been the most common cause of epidemic meningoencephalitis [18], and human disease has been reported in 48 states. Outbreaks of the disease are more common in summer months and early fall.

## 7. Diagnosis

The diagnosis of neuroinvasive disease in WNV infection is based on several factors like exposure to the vector, living in an endemic area, and the clinical presentation. In endemic areas the diagnosis should be considered, especially in the summer [3]. The diagnosis is confirmed with serological studies (WNV antigen-specific enzyme-linked immunosorbent assay) [19, 20].

The test can be performed in the serum or CSF aiming to detect WNV specific antibody. There has been reported cross-reactivity in patients infected with or vaccinated for other flaviviruses [21]. The serology for WNV can be negative in the initial presentation but turns out positive after 8 days of symptom onset in 95% of CSF specimens and 90% of serum specimens [22]. Based on this, in patients with high suspicion for WNV infection, the serology test should be repeated if initially negative. Polymerase chain reaction testing of the serum or CSF is not recommended for the diagnosis of WNV infection in immunocompetent individuals since the peak of viremia happens 3-4 days before initiation of symptoms. The sensitivity of polymerase chain reaction for WNV is low in the CSF and serum (57% and 14%, resp.) [23]. Sensitivity may be higher in immunosuppressed patients with impaired antibody response and prolonged viremia [7]. The CSF findings in patients with neuroinvasive disease are nonspecific and include pleocytosis (neutrophil or lymphocyte predominance) with elevated protein and normal glucose levels [24].

Imaging studies like MRI are also unspecific and data from different series are nonconsistent. In one series, the MRI findings were unremarkable while in other series up to 70% of the patients had abnormalities in the MRI [25–27]. In our paper, both patients presented with encephalitis, which was a diagnostic challenge since all the tests for the usual agents causing encephalitis were negative. The initial CSF studies were not diagnostic, revealing only pleocytosis in both cases. In the first case, MRI showed nonspecific signal in the middle cerebellar peduncles but in the other case MRI was not feasible to obtain because of intracardiac hardware. The diagnosis was made in both cases after correlation with the risk factors, season, symptoms, and laboratory findings.

## 8. Treatment

Currently, there is no specific therapy for WNV infection. The treatment is based on supportive care. Interferon and ribavirin have shown some promising results in vitro but have not been helpful in humans [28]. Interestingly, anti-WNV immunoglobulin has been effective in murine models in Israeli studies where the disease is endemic [29, 30].

## 9. Conclusion

Neuroinvasive WNV infection is associated with significant morbidity especially in the elderly population. High index of suspicion should be maintained in patients with the antecedent event of outdoor activities who present to the ICU with encephalitis or aseptic meningitis of unknown etiology especially in summer months where the disease is more likely to be transmitted by mosquitoes. More studies should be done to determine which factors will predict or be associated with a more severe disease requiring critical care.
